# Tumor Glycosylation: A Main Player in the Modulation of Immune Responses

**DOI:** 10.1002/eji.202451318

**Published:** 2025-03-12

**Authors:** Ernesto Rodriguez

**Affiliations:** ^1^ Amsterdam UMC location Vrije Universiteit Amsterdam Molecular Cell Biology and Immunology Amsterdam The Netherlands; ^2^ Cancer Center Amsterdam Cancer Biology and Immunology Amsterdam The Netherlands; ^3^ Amsterdam Institute for Infection and Immunity Cancer Immunology Amsterdam The Netherlands

**Keywords:** cancer, glyco‐code, glycosylation, lectin receptors

## Abstract

Tumor immune escape refers to the process by which cancer cells evade detection and destruction by the immune system. Glycosylation, a post‐translational modification that is altered in almost all cancer types, plays a crucial role in this process by modulating immune responses. This review examines our current understanding of how aberrant tumor glycosylation contributes to a tolerogenic microenvironment, focusing on specific glycosylation signatures—fucosylation, truncated O‐glycans, and sialylation—and the immune receptors involved. Additionally, the clinical significance of tumor glycosylation is discussed, emphasizing its potential in developing novel therapeutic approaches aimed at improving immune system recognition and targeting of cancer cells. The review underscores the importance of ongoing research in this area to identify effective strategies for countering tumor immune escape and enhancing the efficacy of cancer treatments.

## Graphical abstract

Cancer cells present aberrant glycosylation patterns that contribute to the evasion of antitumor immunity by triggering lectin receptors. Therefore, tumor glycosylation represents an interesting target for novel immunotherapy strategies. This review discusses the different mechanisms by which tumor‐derived glycans induce tolerogenic programs in immune cells and their clinical significance.

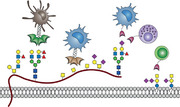

## 1 Introduction

Tumor immune escape is a process by which cancer cells evade detection and destruction by the immune system [1, 2]. During cancer progression, genetic and epigenetic changes increase tumor heterogeneity, fostering a selection process of malignant cells under the pressure of the immune system and the microenvironment [1, 2, 3, 4]. The end product is a complex and heterogeneous disease that presents a plethora of different genotypes and phenotypes but is characterized by the intrinsic immune suppressive nature of most established tumors [2]. Alterations in multiple biological mechanisms contribute to the induction of a tolerogenic microenvironment, including the increased expression of immune checkpoint molecules such as PD‐L1 and CTLA4, secretion of diverse modulatory cytokines like IL‐10 and TGF‐β, metabolic reprogramming, among others [1, 2, 5].

One of the processes that is altered in virtually every cancer type is glycosylation: the enzymatic process that orchestrates the biosynthesis, modification, and degradation of carbohydrate structures, also called glycans, present in free form or covalently attached to proteins, lipids, and RNA [6, 7, 8]. Research has extensively shown that malignant transformation is associated with alterations in glycosylation pathways that directly contribute to critical hallmarks of cancer, including the modulation of the immune system [7, 9, 10]. This review explores our current understanding of how tumor glycosylation impacts immune cell function, discussing its significance in cancer biology and the design of novel therapeutic approaches.

### 1.1 Glycosylation

Glycosylation is a highly complex and regulated metabolic process that involves multiple proteins and enzymes that build up, degrade, or transport glycoconjugates [11, 12]. As they are present in the endoplasmic reticulum or the Golgi apparatus, most of the membrane and secreted proteins get glycosylated while intracellular trafficking along the secretory pathway, resulting in a cell surface rich in glycan structures, known as the glycocalyx [11].

**FIGURE 1:**
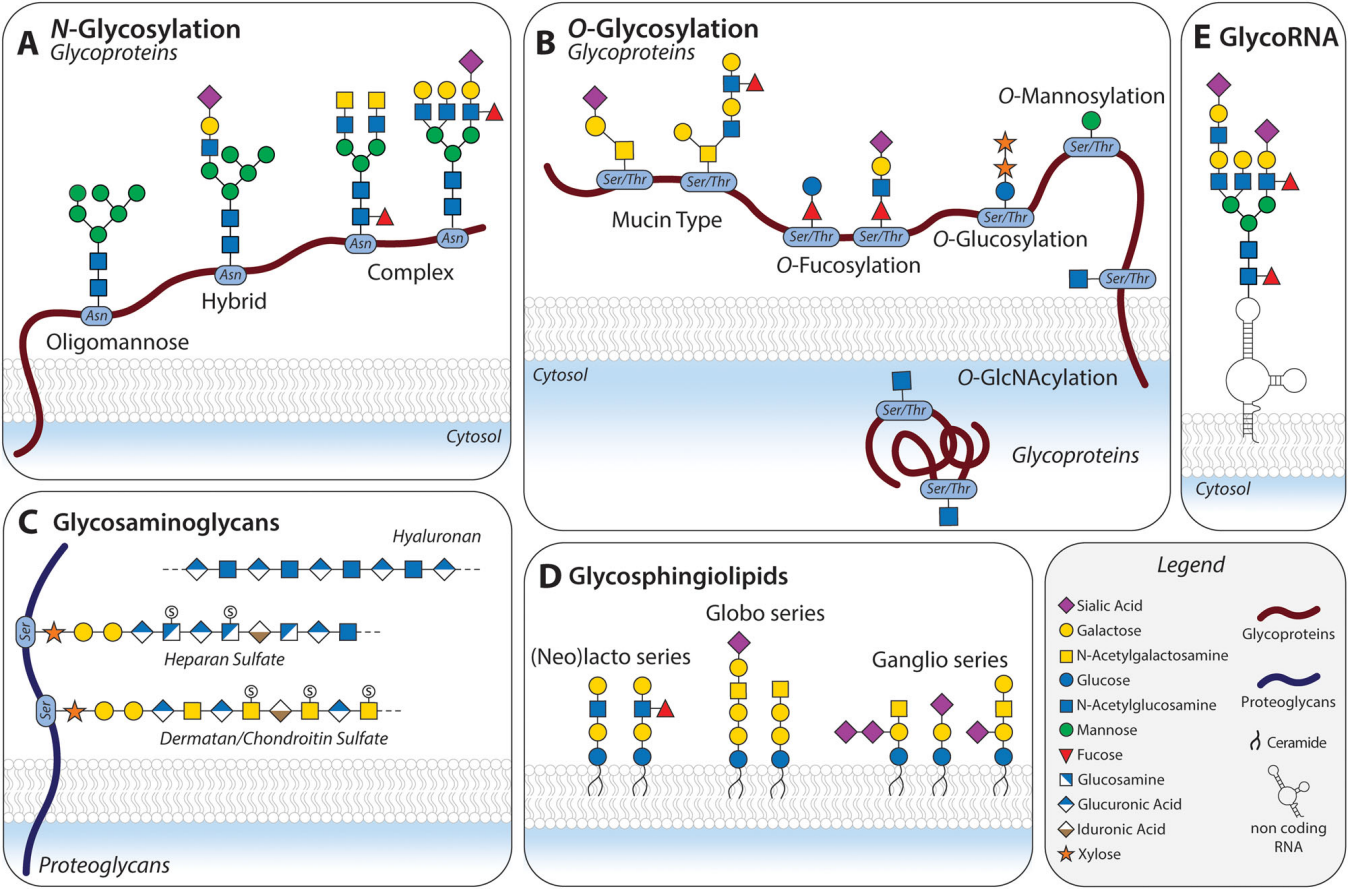
The diversity of the human glycome. The structures found in glycoproteins can be classified according to the linkage between the carbohydrates and the protein in mainly two types: *N*‐ and *O*‐glycosylation (A, B). (A) *N*‐glycans are attached to an asparagine through a residue of *N*‐acetylglucosamine (GlcNAc). They present a core glycan conformed of GlcNAc_2_Man_3_ and branches that can be structurally diverse and can be subsequently classified according to their structure in oligomannose, complex, or hybrid. (B) *O*‐glycans are attached to the hydroxyl group present in the side chain of serine and threonine. The most studied kind of *O*‐glycosylation in cancer is the mucin type, where a residue of *N*‐acetylgalactosamine (GalNAc) is directly linked to the protein. The addition of GlcNAc to a Serine and Threonine present in proteins (*O‐*GlcNAcsylation) is mainly present intracellularly. Other types of *O*‐glycans (*O*‐Fucosylation, *O*‐Mannosylation, *O*‐Glucosylation) are generally present in specific proteins or domains. (C) Proteoglycans are proteins that are present in their structure glycosaminoglycans, covalently bound through a Xylose. Glycosaminoglycans are linear polysaccharides that are classified as heparan sulfate, dermatan/chondroitin sulfate, and hyaluronan. The latter, unlike other GAGs, is present as a free molecule. (D) Glycosphingolipids (GSLs) are glycolipids that contain ceramide as the lipidic component. Distinct biosynthetic pathways lead to the synthesis of three classes of GSLs, denominated (neo)lacto, Globo, and Ganglio series. Gangliosides, negatively charged GSLs containing sialic acid, are an important group of structures in cancer. (E) Recently, it was described the presence of glycosylated noncoding RNA in the cell membrane.

In humans, it involves mainly nine monosaccharides as building blocks, which can be linked together through glycosidic bonds that, unlike proteins, can occur in various positions and orientations, resulting in a diverse array of carbohydrate structures (Figure 1). This complexity is intrinsically linked to the functional diversity of glycans, which are involved in a myriad of biological processes such as quality control, folding and stability of proteins; modulating receptor multimerization and signaling; and mediating cell‐to‐cell interactions, among others [7, 10]. They also carry biological information that can be decoded by carbohydrate‐binding proteins, also called lectins, presenting diverse roles in health and disease, including cell‐to‐cell interaction and cell signaling [13].

An illustrative example of the critical role of glycosylation, particularly relevant to this review, can be observed in the immune system, where it influences cell development and function. Proper glycosylation of T cells is essential for early lineage commitment, as thymic selection and differentiation [14, 15]. Similarly, B cell development is modulated by specific glycosylation pathways that regulate precursor survival and signaling [15, 16]. In antibodies, glycans modulate their interaction with Fc receptors and complement proteins, influencing immune effector functions such as antibody‐dependent cellular cytotoxicity (ADCC) and complement activation [17]. Additionally, immune cells express lectin receptors enabling them to interact with host‐ and pathogen‐derived glycan structures, contributing to self/nonself discrimination [7, 13].

### 1.2 The Tumor Glyco‐code

The first reports that suggest the presence of glycosylation changes in cancer patients are dated in the late 1940s [18, 19, 20, 21]. However, only in recent decades have we started to understand better their role in cancer progression, thanks to the advances in analytical and biological tools. It is now well‐established that the array of glycan structures found in tumor tissue—collectively referred to as the tumor glyco‐code—is fundamentally different from those in healthy tissue [7, 9]. This aberrant glycosylation can arise from alterations in the expression levels of glycosylation‐related genes [22], the functionality of chaperones [23], the localization of enzymes within the secretory pathway [24], and metabolic pathways [25, 26]. In fact, several glycans and glycoproteins with biomarker potential have been identified (Table 1). That is the case of the carbohydrate antigen 19‐9 (CA19‐9), which recognizes the structure sialyl Lewis A (sLe^A^) and is still used today in the clinic as a biomarker for gastrointestinal malignancies [10, 27].

**TABLE 1:** Glycosylation structures and glycoproteins that serve as tumor biomarkers.

Biomarker	Glycan/glycoprotein	Cancer Type	Refs.
CA19‐9	Sialyl‐Lewis A	Pancreatic, gastric	[27, 97]
CA72‐4	Sialyl‐Tn antigen	Gastric, pancreatic	[97, 98]
CA125	MUC16	Ovarian, pancreatic	[27, 99]
CA15‐3	MUC1	Breast	[100]
AFP‐L3	Core‐fucosylation in alpha‐fetoprotein (AFP)^a^	Hepatocellular carcinoma	[101]

^a^Detected based on the recognition by the lectin *Lens culinaris* agglutinin (LCA).

As immune cells can sense changes in the surrounding glycome through a variety of lectin receptors, aberrant glycosylation in cancer reshapes its interactions with the immune system, allowing the formation of new connections that contribute to the development of a tolerogenic microenvironment [7]. This review focuses on the contribution to cancer immune escape of specific glycosylation signatures: fucosylation, truncated O‐glycans, and sialylation. However, other glycosylation pathways, such as glycosaminoglycans—key components of the extracellular matrix—are also known to play a role in cancer progression. These pathways, though mentioned in Figure 1, are beyond the scope of this review and have been thoroughly reviewed previously [28].

### 1.3 Tumor Fucosylation

**FIGURE 2:**
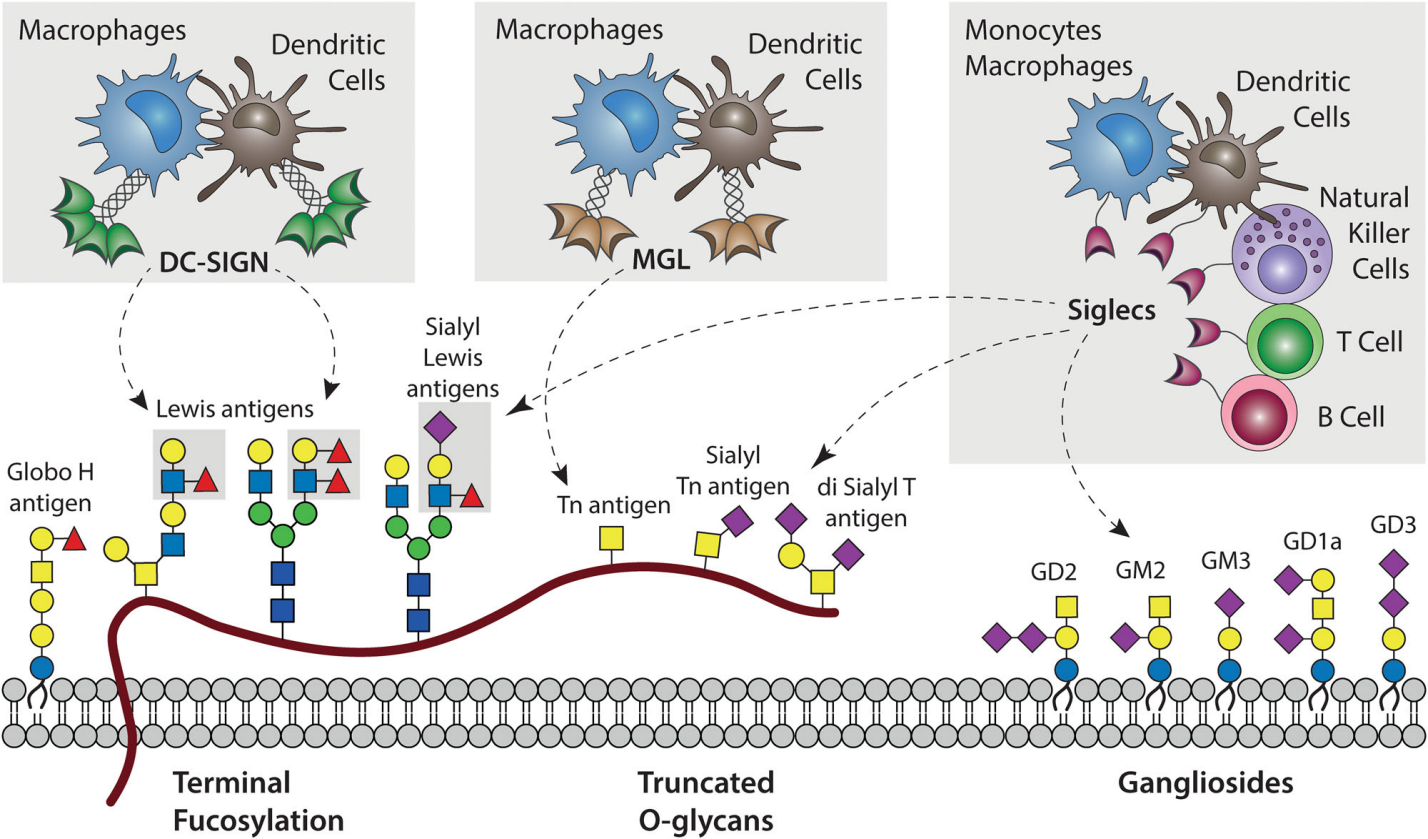
Glycosylation structures enriched in cancer and their interaction with immune cells via lectin receptors.

Fucose‐containing glycans can be divided into three types of structures: core fucosylation, a single fucose linked to the first GlcNAc of *N*‐glycans; *O*‐fucosylation, in which the glycans are bound to the peptide backbone by fucose; and terminal fucosylation, that includes Lewis antigens (Figures 1 and 2). Each of these structures can distinctly influence biological processes and immune responses.

Core fucosylation, catalyzed by the enzyme *FUT8*, can stabilize various proteins on the membranes of cancer cells, including the inhibitory receptors B7‐H3 and PD‐L1 [29, 30]. In this context, fucosylation inhibitors have been shown to enhance the response to immune checkpoint blockade (ICB) therapies [29, 30]. Conversely, a recent report suggests that core fucosylation stabilizes HLA‐DRB1 in a mouse model of melanoma, enhancing ICB efficiency [31]. Therefore, the role of core fucosylation in modulating immune responses may vary across cancer types, influenced by their distinct strategies that contribute to immune escape. Protein *O‐*fucosyltransferase 1 (*POFUT1*) catalyzes the *O*‐fucosylation of EGF repeats present in various membrane proteins, such as Notch receptors, affecting their capacity to interact with their ligands [32]. Terminal fucosylation can serve as ligands for several C‐type lectin receptors (CLRs), a heterogeneous family of Ca^++^‐dependent glycan‐binding proteins that contain soluble and transmembrane receptors. *DC‐SIGN* (dendritic cell‐specific ICAM‑3‑grabbing nonintegrin, CD209) is a CLR present in macrophages and dendritic cells that is able to recognize not only fucosylated glycans but also high‐mannose structures, which trigger inhibitory or activating programs in myeloid cells, respectively [33, 34, 35]. Indeed, fucose‐bearing structures can induce the upregulation of the anti‐inflammatory cytokines (such as IL‐10 and IL‐27), and the differentiation of T cell phenotypes less efficient for antitumoral immunity, such as Th2 and Treg [33, 34, 35]. In tumors, DC‐SIGN is expressed in tumor‐associated macrophages (TAMs) and can interact with Lewis antigens present in epithelial cells, modulating macrophage activation by TLR ligands [7, 35]. However, the lack of a clear functional murine ortholog of DC‐SIGN hinders the study of their physiological role in vivo [7, 36]. Mice have eight homologs of DC‐SIGN, all displaying different glycan specificity and/or cell distribution than those found in humans [36]. Selectins, another type of CLR, can recognize terminal fucosylated structures, with their ligands being the sialylated form of Lewis antigens [37]. The family comprises three members named for their expression: P‐selectin on platelets, E‐selectin on endothelial cells, and L‐selectin on leukocytes.

### 1.4 Truncated O‐glycans

A common feature of various cancer types is the expression of truncated mucin‐type O‐glycans, a product of incomplete glycosylation, instead of fully elongated and complex glycan chains that can be found in healthy tissues (Figure 2) [9]. As a consequence, mucins and proteins with mucin‐like domains, that contain multiple O‐glycosylation sites, are heavily decorated with immature structures, some of which can mediate the interaction with the immune system. The Macrophage Galactose Lectin (MGL, CLEC10A, CD301) is a CLR expressed in macrophages and conventional dendritic cells 2 (cDC2s) that can bind glycans that have terminal N‐acetyl galactosamine (as the Tn antigen) and its signaling modulate cell activation [38, 39]. In fact, the expression of MGL ligands is associated with lower survival in colorectal and cervical cancers [40, 41]. Moreover, several truncated O‐glycans are also decorated by sialic acid (as Sialyl Tn and di‐Sialyl T antigens; Figure 2), giving rise to structures that can trigger the inhibitory receptors Siglecs (Sialic acid‐binding immunoglobulin‐type lectins), which are discussed in the next section. For example, the small *O*‐glycan tetrasaccharide di‐sialyl T antigen present on CD43 is the structure recognized by Sgilec‐7 on leukemia cells [42, 43].

### 1.5 Sialic Acid‐Siglec Axis in Cancer

The surface of most cells in healthy tissues is decorated with glycans containing terminal sialic acid, which the immune system recognizes as self‐structures, and consequently, they have been defined as self‐associated molecular patterns (SAMPs) [44]. In humans, the Siglec family consists of 14 sialic acid‐binding receptors that can recognize sialylated structures expressed on the same cell (cis interaction) or in a neighboring cell (trans interaction) [45]. They are expressed in a broad range of immune cells, including NK, myeloid cells, and B and T lymphocytes [7]. Many Siglecs feature an immunoreceptor tyrosine‐based inhibitory motif in their intracellular domain, and their signaling pathway involves the SHP1 and SHP2 phosphatases, suppressing immune cell activation in a similar fashion to the triggering of PD‐1 by PD‐L1 [7, 45].

Malignant transformation is associated with an overexpression of Siglec ligands that contribute to the tolerogenic microenvironment [7, 46]. In recent years, several research groups have used animal models to show that the removal of cancer sialic acid changes the immune landscape of the tumor microenvironment (TME), leading to increased infiltration of T cells and a better response to ICB [47, 48, 49, 50]. This effect is associated with Siglec‐mediated repolarization of the myeloid compartment in the TME [48, 50]. Triggering of Siglec‐9 in human monocytes by tumor or stromal‐derived sialylated structures favors their differentiation toward TAMs that express CD163 and CD206 [51, 52, 53]. Sialylated structures can also inhibit cytotoxicity mediated by NK cells by triggering the Siglec‐7 and Siglec‐9 receptors [46]. While Siglecs are generally absent from human T cells, recent studies have indicated that Siglec‐9 expression can be induced in the tumor microenvironment, leading to the suppression of T cell activation upon interaction with sialylated glycans [54, 55]. Taken together, these observations highlight the significance of the sialic acid‐Siglec axis in dampening antitumor immune responses, making it a valuable target for the design of new immunotherapy approaches.

### 1.6 Cancer Immunotherapy Meets Glycobiology

Given their role in modulating the immune system and their scarce expression in healthy tissue, the tumor glyco‐code can be seen as neoantigens that represent interesting targets for therapeutic and diagnostic approaches. Beyond specific glycan structures, cancer‐associated glycoforms of proteins are also of value in this context. In the last decades, antitumoral immunotherapy approaches have been developed to target tumor glycosylation, some of which are discussed below.

#### 1.6.1 Tumor Vaccines

Immunization strategies targeting glycans are the foundation for several widely used vaccines against different pathogens [56, 57]. However, this process is challenged by the inherently low immunogenicity of glycan structures, which are T‐independent antigens [57]. Moreover, in the case of tumor‐associated glycans, overcoming immune tolerance is essential, as these structures can be present during embryogenesis or at low levels in healthy tissues [56]. Since glycans alone do not elicit strong T‐cell responses, they are often conjugated to an immunogenic carrier, such as the keyhole limpet hemocyanin (KLH) [58]. Specific antibody response to a vaccine targeting the glycan Globo H correlated to progress‐free survival in patients with metastatic breast cancer in a phase II clinical trial [59]. In neuroblastoma, a vaccine against the gangliosides GD2/GD3 induces robust antibody production when combined with β‐glucan, observing an improved survival that is associated with the levels of GD2‐specific IgG1 [60]. Theratope, a vaccine targeting the sTn antigen developed by Biomira, induces a strong antibody response but showed no benefit in metastatic breast cancer in a phase III clinical trial [61]. However, the expression of sTn before was not evaluated during patient selection, which may explain this outcome.

#### 1.6.2 Glycans for Targeting

Carbohydrate structures can also be used as targeting molecules for vaccine delivery to different APCs, given their unique expression of lectin receptors [62]. For example, ganglioside‐decorated liposomes can specifically target Siglec‐1 expressing cells, as marginal zone macrophages and AXL DCs, and induce T cell activation [63, 64]. Similarly, targeting of DC‐SIGN has been shown to facilitate internalization, cross‐presentation, and the development of tumor‐specific T‐cell responses [65, 66]. Recently, Tumor Immune Cell Targeting Chimera (TICTACs) were developed by the group of Prof. Carolyn Bertozzi, in which antibodies specific for immune checkpoint are conjugated to a ligand for CD206, a TAM marker [67]. Engagement of this receptor induces the internalization of the complex CD206‐TICTAC‐immune checkpoint, effectively removing them from the TAM surface [67]. Altogether, these results highlight the potential role of glycan structures for the specific targeting of immune cells.

#### 1.6.3 Anti‐glycan Antibodies

Following the advent of monoclonal antibody (mAb) technology in the 1970s, numerous research groups aimed to develop cancer‐specific mAbs, leading to the generation of various antibodies targeting tumor‐associated glycans, some of which serve as the base for tools that are currently used in the clinic [56, 68–73]. Dinutuximab, a mAb specific for the ganglioside GD2, is a humanized version of one of those early generated clones, and is able to induce antibody‐dependent cell‐mediated cytotoxicity (ADCC) and complement‐dependent cytotoxicity (CDC) toward neuroblastoma cells [73, 74]. It was approved by the FDA for the treatment of high‐risk pediatric neuroblastoma, showing improved survival rates in Phase III clinical trials [56, 75]. The biomarker CA19‐9, corresponding to sialyl Lewis A, was initially discovered as the structure recognized by the clone 19–9 [68, 70]. A fully human mAb specific for CA19‐9, developed from patients immunized with sLe^A^‐KLH, is capable of inducing ADCC and CDC in cancer cells and is currently being evaluated as a therapeutic agent or imaging tool for malignancies that express this antigen, as pancreatic cancer [56, 76, 77]. Several clinical trials are ongoing to study the safety and efficacy of anti‐glycan antibodies in the treatment of different malignancies [56].

#### 1.6.4 CAR Immune Cells

Immune cells (mainly T and NK cells) can be engineered to express chimeric antigen receptors (CARs) that can redirect their specificity to a tumor target of interest, induce their activation, and lead to cell death. Therapies based on CAR‐T cells have revolutionized the treatment of hematological malignancies [78]. The use of CAR‐T cells targeting a glycoform of MUC1 containing the Tn antigen was effective in preclinical models of leukemia and pancreatic cancer, and clinical trials are currently ongoing [79]. In neuroblastoma, GD2‐CAR T cells have shown promising results in a phase I clinical trial [80, 81, 82]. The targeting domain in most CARs is based on single‐chain variable fragments (scFv) derived from monoclonal antibodies [78]. The use of carbohydrate‐recognition domain from lectins as an alternative for the generation of CARs targeting glycan structures has been recently proposed [83, 84]. Future research is needed to better understand their value in a clinical setting.

#### 1.6.5 Remodeling the Glycocalyx

Given their role in inducing a tolerogenic TME, the removal of modulatory tumor‐associated glycans can improve anticancer immune responses. Chemical inhibitors that abrogate sialylation pathways reduce tumor burden and increase T‐cell responses in a mouse model of melanoma (B16F10) [85, 86]. On the other hand, the fucosylation inhibitor 2‐fluorofucose showed promising results in both in vitro and in vivo studies, but its Phase I clinical trial was terminated due to safety concerns [87, 88]. As glycosylation also plays a role in healthy tissues, it is necessary to improve the specific delivery to the tumor as a way to avoid potential side effects.

Conjugates of tumor‐targeting antibodies with neuraminidases, enzymes that cleave terminal sialylation, are effective tools for the remodeling glycocalyx in the TME, leading to delayed tumor growth, the repolarization of TAMs and an enhanced efficacy of ICB [47, 48]. This strategy can be adapted to the use of other relevant glycosidases or enzymes that can modify the tumor glyco‐code, as is the case of bacterial mucinases, which can cause proteolysis of cancer‐associated mucins [56, 89].

## 2 Conclusion and Future Perspectives

This review highlights extensive literature showing the critical role of the tumor glyco‐code in immune system modulation, which has garnered increased recognition from the scientific community, and significant advancements are anticipated in the coming years. Analytical methods have had great progress in recent years, enabling the identification of glycosylation sites within proteins, detailed glycan structure differentiation, and high throughput analysis [90]. However, most of these techniques require specialized equipment and knowledge that is not within reach of every research group and, even less, the clinic.

Bridging the gap between glycobiology and other research and clinical fields requires developing innovative tools that facilitate the study of the tumor glyco‐code. Despite that some anti‐glycan mAbs are available, the generation of new ones is challenging given the limited immunogenicity of carbohydrate structures, frequently leading to low‐affinity mAbs of IgM isotype that cross‐react with related glycans [91, 92]. Alternative systems have been used in recent years for the generation of highly specific anti‐glycan probes, such as the immunization of lampreys (*Petromyzon marinus*) and llamas (*Lama glama*) [92, 93]. Directed evolution can further enhance the specificity of these reagents [94]. Therefore, it is expected that new glycobiology tools will be developed in coming years, which will facilitate the detection of specific glycan structures in different contexts.

Finally, as the oncology field is moving toward single‐cell and multi‐omics technologies, it is important to find new methods able to integrate also glycomic information. Plant lectins have been used for the analysis of glycans in different single‐cell technologies, such as mass cytometry and scRNA‐Seq [95, 96]. As more specific anti‐glycan probes are developed, we can expect more detailed information about the tumor glyco‐code and its relationship with immune cells at a single‐cell level.

## Conflicts of Interest

The authors declare no conflicts of interest.

### Peer Review

The peer review history for this article is available at https://publons.com/publon/10.1002/eji.202451318

## Data Availability

Data sharing is not applicable to this article as no datasets were generated or analyzed during the current study.
